# Supplementary material for: An indicator cell assay for blood-based diagnostics
Source: PLoS One. 2017 Jun 8;12(6):e0178608. doi: 10.1371/journal.pone.0178608 (PMC5464608; doi:10.1371/journal.pone.0178608)
Supplement: S1 Fig — (DOCX) [file pone.0178608.s001.docx]

**ROC curves**

**Alzheimer’s Disease Classifier Performances**


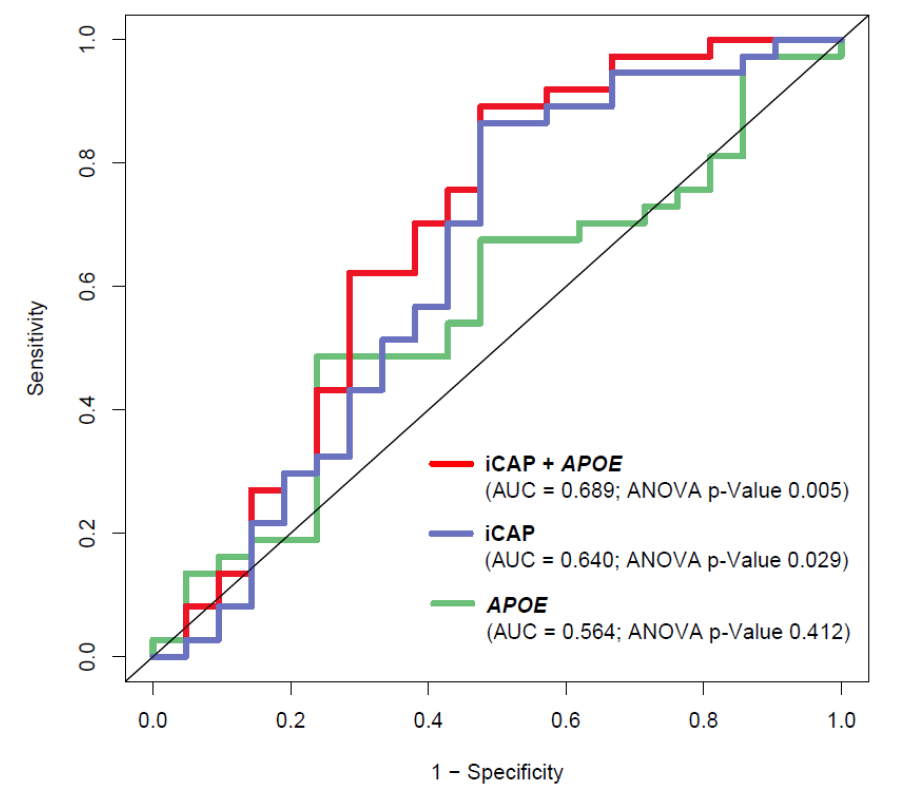


**Supplementary Figure S1.** ROC curves for iCAP-*APOE4* AD classifier (iCAP classifier with *APOE4* count included as a feature), the iCAP AD classifier, and the *APOE4* classifier.
